# Prefrontal cortex miR-29b-3p plays a key role in the antidepressant-like effect of ketamine in rats

**DOI:** 10.1038/s12276-018-0164-4

**Published:** 2018-10-29

**Authors:** Yun-Qiang Wan, Jian-Guo Feng, Mao Li, Mao-Zhou Wang, Li Liu, Xueru Liu, Xiao-Xia Duan, Chun-Xiang Zhang, Xiao-Bin Wang

**Affiliations:** 1 Department of Anesthesiology, The Affiliated Hospital of Southwest Medical University, Luzhou, Sichuan Province People’s Republic of China; 2 Laboratory of Anesthesiology, The Affiliated Hospital of Southwest Medical University, Luzhou, Sichuan Province People’s Republic of China; 3 Department of Intensive Care Unit, The Affiliated Chaoyang Hospital of Capital Medical University, Beijing, People’s Republic of China; 4 Department of Biomedical Engineering, School of Medicine, University of Alabama at Birmingham, Birmingham, AL USA

**Keywords:** Cognitive neuroscience, Psychiatric disorders

## Abstract

Ketamine has a rapid, obvious, and persistent antidepressant effect, but its underlying molecular mechanisms remain unknown. Recently, microRNAs (miRNAs) have emerged as important modulators of ketamine’s antidepressant effect. We investigated the alteration in miR-29b-3p in the brain of rats subjected to ketamine administration and chronic unpredictable mild stress (CUMS), and a sucrose preference test and forced swimming test were used to evaluate the rats’ depressive-like state. We used recombination adeno-associated virus (rAAV) or lentivirus-expressing miR-29b-3p to observe the change in metabotropic glutamate receptor 4 (GRM4). Cell culture and electrophysiological recordings were used to evaluate the function of miR-29b-3p. Ketamine dramatically increased miR-29b-3p expression in the prefrontal cortex of the normal rats. The dual luciferase reporter test confirmed that GRM4 was the target of miR-29b-3p. The miR-29b-3p levels were downregulated, while the GRM4 levels were upregulated in the prefrontal cortex of the depressive-like rats. The ketamine treatment increased miR-29b-3p expression and decreased GRM4 expression in the prefrontal cortex of the depressive-like rats and primary neurons. By overexpressing and silencing miR-29b-3p, we further validated that miR-29b-3p could negatively regulate GRM4. The silencing of miR-29b-3p suppressed the Ca^2+^ influx in the prefrontal cortex neurons. The miR-29b-3p overexpression contributed to cell survival, cytodendrite growth, increases in extracellular glutamate concentration, and cell apoptosis inhibition. The overexpression of miR-29b-3p by rAAV resulted in a noticeable relief of the depressive behaviors of the CUMS rats and a lower expression of GRM4. The miR-29b-3p/GRM4 pathway acts as a critical mediator of ketamine’s antidepressant effect in depressive-like rats and could be considered a potential therapeutic target for treating major depression disorder.

## Ketamine: Insights into antidepressant effects

Changes in the levels of a microRNA molecule that regulates the activity of a specific gene may be responsible for some of the antidepressant effects of the drug ketamine. Ketamine is used as a tranquilizer and anesthetic, especially in veterinary practice, but there is increasing interest in its antidepressant action in humans. Researchers in China and USA led by Xiao-Bin Wang at the Affiliated Hospital of Southwest Medical University in Luzhou, China, found that ketamine dramatically raised the levels of a specific microRNA in the prefrontal cortex region of rats’ brains. This effect was linked to the ability of the microRNA to inhibit a gene and thus decrease the production of a receptor protein in brain cell membranes. The molecular signaling pathway this research has identified could be a target for new antidepressant therapies.

## Introduction

Major depressive disorder (MDD) is a common chronic mood disorder that results in heavy social and economic burdens^1^. By 2020, MDD is expected to be the second leading cause of disability worldwide^2^. However, to date, the prospect of antidepressant treatment is poor. In addition, traditional antidepressant drugs require a few days or weeks to achieve their antidepressant effect. The main reason for these inadequacies in the treatment of depression is that a comprehensive understanding of the pathogenesis of depression is lacking. Ketamine is a *N*-methyl-d-aspartate receptor antagonist. Accumulating evidence from clinical and basic research shows that ketamine exerts a quick and persistent antidepressant-like effect^3–7^. Compared with traditional antidepressant drugs, the antidepressant effect of ketamine is obvious and quick. Due to its clear and rapid antidepressant effect, ketamine has become a topic of interest in the study of depression. However, the functional mechanism of ketamine still remains unclear.

miRNAs constitute a class of small non-coding RNA molecules whose function is to regulate the transcription and translation of downstream-related genes. Furthermore, recent studies suggest that miRNAs are associated with the pathophysiology of depression and could be potential targets for novel antidepressant treatment^8–11^. The miR-29 family members have been shown to mediate neuropathological processes in response to environmental stress factors^12^ and have been suggested as potential biomarkers for the diagnosis and prognosis of neuropsychiatric disorders^13^ and relevant neurodegenerative diseases^14–17^. In our previous report, the acute administration of ketamine resulted in the abnormal expression of various miRNAs (including those belonging to the miR-29 family), which was involved in the antidepressant effect of ketamine^18^. miR-29b expression is elevated in the hippocampus of early-stress rats after acute ketamine administration^11^. Metabotropic glutamate receptor 4 (GRM4) has been predicted to be the target gene of miR-29 in bioinformatic analyses. GRM4, which belongs to metabotropic glutamate receptor group III, is mainly expressed in presynaptic membranes of brain neurons. The main function of GRM4 is regulating dopamine, glutamate, tryptophan, and neurotransmitter metabolism, thus affecting neurotransmitter release-mediated neural signaling pathways^19^. In addition, relevant studies have shown that the increased expression of GRM4 is closely related to the occurrence and development of depression; hence, GRM4 may be involved in the pathogenesis of depression^20^.

In this study, we investigated the effect of ketamine on miR-29a/b/c-3p expression in the hypothalamus, hippocampus, and prefrontal cortex (PFC) of depressed rats and the subsequent regulation of GRM4 expression. Our findings may provide potential therapeutic target genes and open up novel avenues for drug design for MDD treatment.

## Materials and methods

### Ethics statement

All experimental procedures involving animals were performed in accordance with the NIH Guide for the Care and Use of Laboratory Animals and the Chinese Society for Neuroscience and Behavior Recommendations for Animal Care. The experimental surgeries and treatments were approved by the Ethics Committee of the Affiliate Hospital of Southwest Medical University (approval number Ky2012044).

### Subjects and drug treatment

Adult male Sprague–Dawley rats (150–200 g) were purchased from Chengdu Dashuo Animal Center. The rats were acclimated to the normal experimental environment (12 h light/dark cycle, 22 ± 2 °C room temperature) for 1 week with food and water available ad libitum. The rats received intraperitoneal injections of 10 mg/kg ketamine, which is the dose found by previous studies to be appropriate for producing an antidepressive effect in rats^21,22^. The control rats received intraperitoneal administrations of the same volume of saline. After 24 h, the rats were sacrificed for the miRNA and protein analyses.

### Chronic unpredictable mild stress

The rats were subjected to chronic unpredictable mild stress (CUMS) as previously described^23^. Briefly, the CUMS procedure used in this study involved the following 14 mild stressors: continuous overnight lighting, tilted cage, water deprivation, food deprivation, noise, wet padding, strange objects, no padding, suspension (6 h), tail pinch, combined cages, shaker stress, exchanging rat cages, and changing the feeding environment. The stressors were randomly performed every day for 35 days, but the same stressor was never applied on two consecutive days. The rats in the control group were housed separately in another room. Depression-related behavioral tests, including the sucrose preference test and forced swimming test (FST), were performed on days 28 and 35 after CUMS. The weight and fur scores were also evaluated as described by Ducottet et al^24^.

### Sucrose preference test

The sucrose preference test was performed using the method described by Sergio et al^25^. The data were collected on days 0, 28, and 35 after CUMS. Briefly, the rats were habituated to drink 1% sucrose solution from two bottles for 24 h. After fasting and water deprivation for the following 24 h, the rats were allowed to drink one bottle of 1% sucrose solution and another bottle of water freely for 3 h. The water and sucrose consumption were measured, and the preference for sucrose over water (sucrose/[sucrose + water] × 100%) was used as a measure of the reduced ability to experience pleasure.

### Forced swimming test

The FST was conducted in a cylinder that was 65 cm tall and 30 cm in diameter that was filled 40 cm high with water (22–23°). The FST was performed for 6 min, and the immobility time (in s) was recorded during the final 5 min. The immobility time was defined as the time during which the rat stood still without struggling, used only essential movements to keep its head above water, or contacted the bottom for more than 1 s.

### Cell culture and drug treatment

Primary PFC neuronal cultures were prepared as previously described^26^. Briefly, the PFC was dissected from an embryonic day 16.5 brain, and the neuronal cells were dissociated by trypsin digestion. The cells were seeded in 35 mm poly-lysine-coated plates at a final density of 1 × 10^6^ cells/ml in Neurobasal medium (Gibco) supplemented with B27 and 2 mM GlutaMAX. The cells were maintained in a humidified atmosphere of 5% CO_2_ and 95% air at 37 °C. After 2 h, the Neurobasal medium supplemented with B27 and 2 mM GlutaMAX in the plates was changed to Neurobasal medium supplemented only with B27. The primary cells were allowed to grow for 7 days before treatment and then treated with ketamine at a final concentration of 50 μM for 0, 1, 3, 6, or 12 h. The cells were harvested for the miRNA-29b-3p and GRM4 expression analyses. The rat brain glioma C6 cells and human embryonic kidney 293T (HEK293T) cells were kindly provided by the Stem Cell Bank of the Chinese Academy of Sciences. The C6 cells were maintained in DMEM/F12 medium, and the HEK293T cells were cultured in RPMI-1640 medium. The cells were maintained at 37 °C in a humidified CO_2_ incubator in a medium containing 10% heat-inactivated fetal bovine serum (Biological Industries, CT, USA), 100 U/ml penicillin, and 0.1 mg/ml streptomycin.

### siRNA and miRNA mimic transfection

The small interfering RNA (siRNA)-targeting GRM4 (GRM4-siRNA) and negative control siRNA (NC) were designed and synthesized by GenePharma (China). The rat miR-29b-3p mimic (UUCCCUUUGUCAUCCUAUGCCU) and negative control (UUUGUACUACACAAAAGUACUG) were purchased from RiboBio Co. (China). The cells were transiently transfected with siRNAs or mimics using RNAiMAX Transfection Reagent (Invitrogen, USA) according to the manufacturer’s instructions. The cells were subsequently examined 24 h after transfection.

### miRNA target prediction and luciferase assays

The candidate genes targeted by miR-29b-3p were predicted using the following four miRNA target prediction databases: MicroCosm (http://www.ebi.ac.uk/enright-srv/microcosm/htdocs/targets/v5/), miRanda (http://www.microrna.org/microrna/home.do), TargetScan (http://www.targetscan.org/), and miRDB (http://mirdb.org/miRDB/). The genes that were predicted by all four databases and implicated in the pathophysiology of MDD were selected for a luciferase reporter assay.

A dual luciferase reporter assay system (Promega, USA) was used to determine the direct interaction between miR-29b-3p and GRM4. The primers used to generate the GRM4 wild-type 3′-untranslated region (3′-UTR) containing miR-29b-3p-binding site reporter constructs based on pmirGLO (Promega, USA) were as follows: wtGRM4–3′-UTR, forward 5′-AAACTAGCGGCCGCTAGTGTGGCTTGGTGCTGAGGATT-3′ and reverse 5′-CTAGAATCCTCAGCACCAAGCCACACTAGCGGCCGCTAGTTT-3′. The primers used for the mutant GRM4 3′-UTR reporter constructs were as follows: mutGRM4–3′UTR, forward 5′-AAACTAGCGGCCGCTAGTGTGGCTCATGTACGAGGATT-3′ and reverse 5′-CTAGAATCCTCGTACATGAGCCACACTAGCGGCCGCTAGTTT-3′. For the luciferase assays, C6 cells were transiently co-transfected with 50 nM rno-miR-29b-3p mimics or control mimics and 0.5 µg pmirGLO containing the wild-type or mutated GRM4 3′-UTR. The luciferase activity was analyzed 48 h after transfection as indicated by the manufacturer’s instructions.

### qRT-PCR analysis

The miRNA-29a/b/c-3p and GRM4 mRNA expression levels were determined using quantitative reverse transcriptase polymerase chain reaction (qRT-PCR) using the mirVana qRT-PCR miRNA Kit (Ambion Life Technologies, USA), according to the manufacturer’s instructions. A complementary DNA template generated from 1 μg total RNA was used for the qRT-PCR analysis. The miRNA-29a/b/c-3p and GRM4 mRNA amplification and detection were performed using an ABI 9700 Fluorescence PCR System (Applied Biosystems, USA). The primers used for miRNA-29a/b/c-3p, U6, GRM4, and β-actin in the qRT-PCR analysis are shown in Table 1. The fluorescence signal was normalized to unify the internal reference level, and the threshold cycle (*C*_T_) was set in the exponential amplification phase of the PCR. The relative gene expression of miRNA-29a/b/c-3p and GRM4 mRNA was calculated using the 2^−ΔΔCT^ method.

**Table 1:** Sequences of primers used for the real-time quantitative PCR analysis

Gene	Direction	Primer sequence	Length
*Rno-U6*	F	TGCGGGTGCTCGCTTCGGCAGC	22 bp
	R	CCAGTGCAGGGTCCGAGGT	19 bp
*Rno-miR-29a-3p*	F	TGCGGTAGCACCATCTGAAA	20 bp
	R	CCAGTGCAGGGTCCGAGGT	19 bp
*Rno-miR-29b-3p*	F	TGCGG TAGCACCATTTGAAAT	20 bp
	R	CCAGTGCAGGGTCCGAGGT	19 bp
*Rno-miR-29c-3p*	F	TGCGG TAGCACCATTTGAAA	20 bp
	R	CCAGTGCAGGGTCCGAGGT	19 bp
*Rno-β-actin*	F	AAGTCCCTCACCCTCCCAAAAG	22 bp
	R	AAGCAATGCTGTCACCTTCCC	21 bp
*Rno-GRM4*	F	AGTGACAACAGCCGCTATGAC	21 bp
	R	CACACACCTCCGTTCTCTCG	20 bp

### Western blot analysis

The total protein extracted from the rat cerebral tissue or cultured neurons was used to analyze the GRM4 protein levels via a Western blot analysis. The cerebral tissue and primary neuron cells were lysed using RIPA lysis buffer with phenylmethylsulfonyl fluoride (Beyotime, China). The protein concentration was quantified by a BCA assay (Beyotime, China). Thirty micrograms of total protein from each sample were subjected to 12% sodium dodecyl sulfate-polyacrylamide gel electrophoresis and then transferred to polyvinylidene difluoride (PVDF) membranes (Millipore, USA). The membranes were blocked in TBST (Tris-buffered saline, 0.1% Tween-20) with 5% skim milk at room temperature for 1 h and then incubated with a primary GRM4 antibody (Abcam, USA) at 4 °C overnight. After three washes with TBST buffer, the PVDF membranes were further incubated with a secondary horse radish peroxidase-conjugated antibody. The immunoblotting bands were detected via Western Lightning Chemiluminescence Reagent (Perkin Elmer, USA). An AlphaImager analyzer (Alpha Innotech Corporation, CA, USA) was used to measure and quantify the optical density values of the protein bands.

### Apoptosis assay

Apoptosis in the cultured neurons was measured by terminal deoxynucleotidyl transferase dUTP nick-end labeling (TUNEL) staining as previously described^27^. Briefly, hippocampal neurons cultured on coverslips in 24-well plates were fixed in 4% paraformaldehyde. The TUNEL staining was conducted using an In Situ Cell Death Detection Kit (Roche, USA) according to the manufacturer’s protocol. The number of TUNEL-positive cells was counted under a fluorescence microscope.

### Generation of miR-29b-3p-overexpressing and miR-29b-3p-silencing lentiviruses and cell infection

The miR-29b-3p-overexpressing and miR-29b-3p-silencing lentiviral particles (lent-over/miR-29b-3p and lent-inhib/miR-29b-3p) were purchased from Hanbio Biotechnology Co., Ltd. (China). Lenti-NC served as a negative control. Briefly, pre-miR-29b-3p was amplified from the genome of a C6 cell and cloned into the lentivirus expression vector pHBLV-U6-ZsGreen-Puro (Hanbio Biotechnology Co., Ltd., China) with *Bam*HI and *Eco*RI sites to overexpress miR-29b-3p. The details of pre-miR-29b-3p are shown in Supplemental figure. The antagonist short hairpin RNA (AACACTGATTTCAAATGGTGCTA) was inserted into the lentivirus vector pHBLV-U6-Scramble-ZsGreen-Puro (Hanbio Biotechnology Co., Ltd., China) to silence miR-29b-3p. Twenty multiplicity of infection lentivirus particles were added to the medium of rat PFC primary neuron cells. The infection effects were detected by immunofluorescence microscopy. The cells were analyzed by qRT-PCR and Western blotting 72 h after the lentivirus infection.

### Generation of miR-29b-3p-overexpressing AAV and stereotaxic surgery

The miR-29b-3p-overexpressing adeno-associated virus (AAV) (AAV-miR-29b-3p) was purchased from Hanbio Biotechnology Co., Ltd. (China), and AAV-GFP served as a negative control. Briefly, pre-miR-2b-3p was cloned into the AAV vector pAAV-CMV-ires-hrEGFP with *Bam*HI and *Eco*RI restriction sites to construct the AAV transgene plasmid pAAV-CMV-miR-29b-3p-ires-hrEGFP. pAAV-RC9, pHelper, and the AAV transgene plasmid were co-transfected into HEK293 cells. At 72 h after transfection, the cells were incubated with Benzonase endonuclease (Sigma, USA) at 37 °C for 1 h, and then the AAV particles were purified by a heparin column (Sigma, USA). After the purification, the AAV particles were concentrated using Amicon Ultra-4 (Millipore, USA). AAV was aliquoted and stored at −80 °C. The rats were anesthetized with sodium pentobarbital (60 mg/kg, intraperitoneally (i.p.)) and received injections into the PFC (+4.7 mm AP, ± 2 mm ML, and −1.2 mm DV) using a stereotaxic frame (Kopf Instruments, CA, USA) linked to a digital rat brain atlas. Two microliters of AAV-miR-29b-3p or AAV-GFP mixed with 1 μl of 20% mannitol were injected at a speed of 0.1 μl/min. The behavioral experiments were performed after 4 weeks to allow the rats injected with the AAVs to recover and the miRNA to be adequately expressed. The AAV infection effects were detected by immunofluorescence microscopy. The expression of miR-29b-3p and its target gene GRM4 in the rat PFC tissue was analyzed by qRT-PCR and Western blotting 28 days after the AAV infection.

### Cell viability assay

The dissociated neuronal cells were seeded in poly-d-lysine-coated 96-well plates. The cells were maintained in a humidified atmosphere of 5% CO_2_ at 37 °C for 5 days and then infected with lenti-over/miR-29b-3p, lenti-inhib/miR-29b-3p, or lenti-NC particles. After 72 h, MTT (3-(4,5-dimethylthiazol-2-yl)-2,5-diphenyl tetrazolium bromide) reagent (Sigma, USA) was added to the medium, and the cells were incubated for 4 h before measuring the absorbance at 490 nm.

### Extracellular and intracellular glutamate assay

The extracellular and intracellular glutamate levels were measured using the BioVision’s Glutamate Assay Kit. The dissociated neuronal cells were seeded in poly-d-lysine-coated 6-well plates and cultured for 3 days. Subsequently, the cells were infected with the lenti-over/miR-29b-3p, lenti-inhib/miR-29b-3p, or lenti-NC particles. After 72 h, the cell culture medium was directly diluted in the assay buffer for the extracellular glutamate determination. The cell numbers were normalized by the MTT assay. The cells (1 × 10^6^) were homogenized in 100 μl assay buffer for the intracellular glutamate assay. The glutamate levels were quantified by measuring the optical density at *λ* = 450 nm using a microplate reader. The final glutamate concentration was calculated according to standard curves constructed using known concentrations of exogenous glutamate.

### Calcium-sensitive fluorometric measurements

The calcium imaging experiments were conducted using primary neuronal cells infected with the lenti-over/miR-29b-3p, lenti-inhib/miR-29b-3p, and lenti-NC particles. After three washes with Hanks’ balanced salt solution (HEPES buffer), the cells were treated with 5.0 μM Fura-2 AM (Netherlands) for 60 min at 37 °C in a CO_2_ incubator. The cells were sequentially illuminated (6 s each time for a total of 1 min) at 340 and 380 nm (excitation wavelengths) under a laser scanning confocal microscope (Olympus, Japan). The calcium-dependent fluorescence signals were measured at 510 nm (emission wavelength). The relative intracellular Ca^2+^ level was determined using the formula △*F* = (*F* − *F*_min_)/(*F*_max_ − *F*), where *F* is the fluorescence intensity, *F*_min_ was determined by imaging the infected cells in a calcium-free saline solution, and *F*_max_ was determined by imaging the infected cells in normal saline solution with 5 M ionomycin (Sigma-Aldrich, USA).

### Electrophysiological recordings

The electrophysiological recordings were performed as previously described^18^. Briefly, the external solution used for *I*_Ca_ consisted of (in mM, pH 7.4) choline-Cl 110, TEA-Cl 20, 4-AP 10, MgCl_2_ 1, BaCl_2_ 10, HEPES 10, glucose 10, and TTX 10^−3^. The internal solution used for *I*_Ca_ consisted of (in mM, pH 7.2) CsCl 110, TEA-Cl 30, EGTA 10, HEPES 10, and ATP-Mg3. The primary neuron cells were washed with bathing solution. The suspension of the neuronal cells was plated on the recording chamber of an inverted microscope (Olympus, Japan). A single neuron cell infected with a lentiviral particle was chosen. The whole-cell patch-clamp recording was performed with a patch-clamp amplifier. The acquisition, storage, and analysis of the current signals were performed by a MultiClamp 700B Patch-Clamp Amplifier (Axon Instruments), DigiData 1440A digital analog converter, and pClampex 10. 0 (Axon Instruments). The *I*_Ca (HVA)_ current (inward electric current) was recorded using stepwise 10-mV polarizing pulses (200-ms duration) from a constant holding potential of −50 mV in the cells. The whole-cell patch-clamp recordings of the lentivirus-infected PFC neuron cells were also performed after 30 min of incubation with drugs, including a GRM4 antagonist (MSOP, 150 μM), P/Q-type Ca^2+^ channel blocker (ω-agatoxin TK, 250 nM), N-type Ca^2+^ channel blocker (ω-conotoxin GVIA, 250 nM), R-type Ca^2+^ channel blocker (SNX-482, 100 nM), phospholipase C (PLC) inhibitor (U73122, 1 μM), and protein kinase C inhibitor (GF109203X, 20 nM).

### Statistical analysis

The Statistical Package for the Social Sciences (SPSS) version 16.0(SPSS Inc., Chicago, IL, USA) and GraphPad Prism 5.0 (San Diego, CA, USA) were used for the statistical analyses. All data are presented as the mean ± SEM. Differences between two groups were determined by Student’s *t* test, and multigroup differences were determined by one-way analysis of variance, followed by Tukey’s post hoc test. *P* values <0.05 were considered statistically significant differences.

## Results

### Ketamine increased miR-29b-3p expression in the rat PFC

Recent studies suggest that miRNAs are associated with MDD pathogenesis^28^, and our previous reports show that miR-29a/b/c-3p was probably involved in MDD^29^. Ketamine administration is accepted as a rapid and persistent antidepressant treatment for MDD^22,30,31^. MDD is associated with altered neuronal and structural plasticity and neurogenesis in brain regions, including the hypothalamus, hippocampus, and PFC^32–34^. However, whether miR-29a/b/c-3p responds to ketamine treatment in the hypothalamus, hippocampus, and PFC is still unknown.

**Fig. 1:**
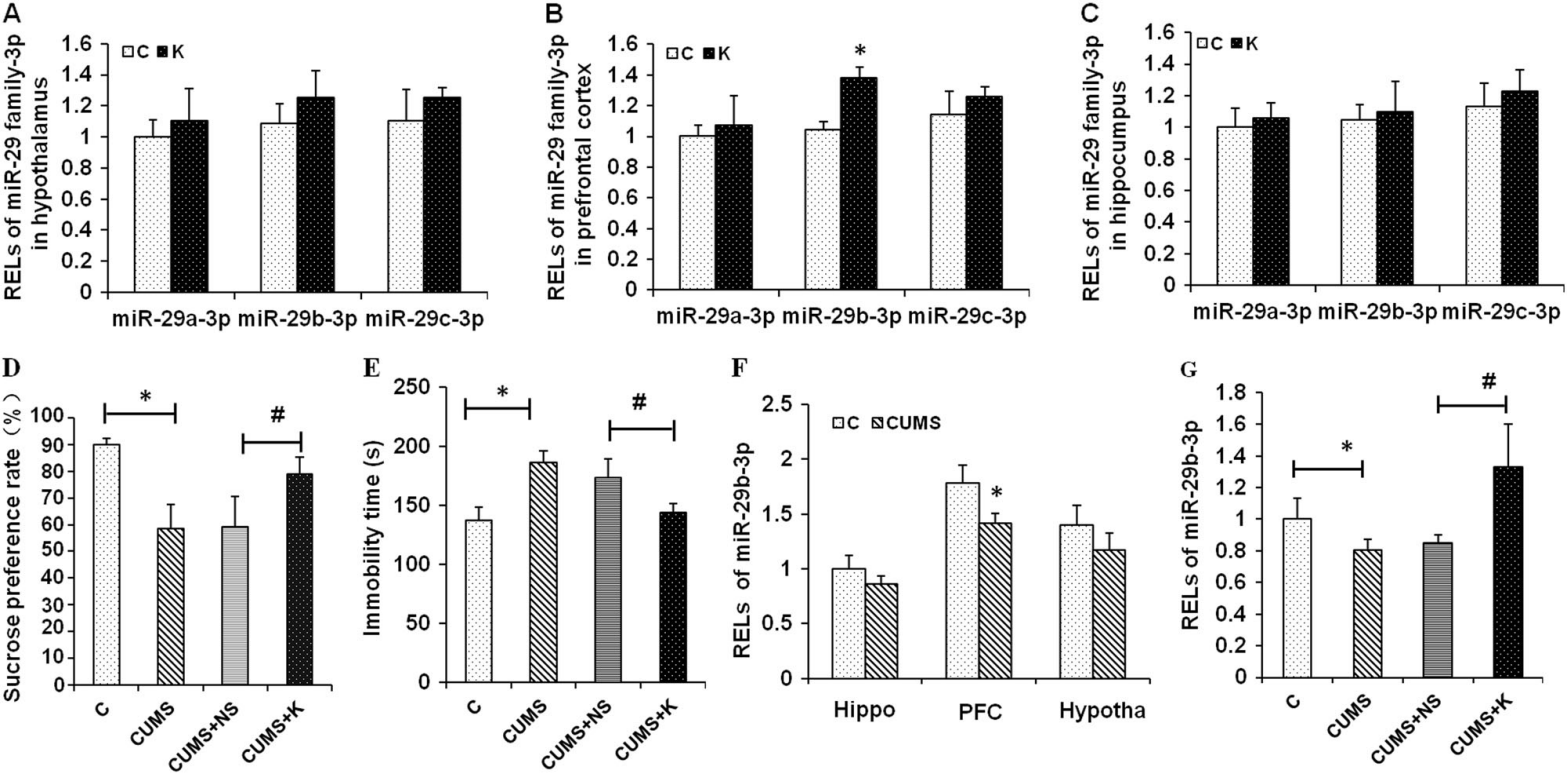
Effects of ketamine on miR-29a/b/c-3p expression in the rat brain. RELs of miR-29a/b/c-3p 24 h after ketamine treatment (10 mg/kg) in the hypothalamus (**a**), prefrontal cortex (**b**), and hippocampus (**c**). The sucrose preference rate (**d**) and forced swimming test (**e**) in rats after 5 weeks of CUMS. RELs of miR-29b-3p in different brain regions of depressed rats (**f**). RELs of miR-29b-3p in the prefrontal cortex of depressed rats 24 h after ketamine treatment (10 mg/kg) (**g**). RELs relative expression levels, Hippo hippocampus, PFC prefrontal cortex, hypotha hypothalamus, C control group, K ketamine group, CUMS chronic unpredictable mild stress, CUMS + NS chronic unpredictable mild stress + 0.9% saline group, CUMS + K chronic unpredictable mild stress + ketamine group (*n* = 8). RELs = 2^−Ct{[(delta experimental sample) Ct-U6 Ct] − [(control group) Ct-U6 Ct]}^. The data represent eight sets of independent experiments and are shown as the means ± SD. *p < 0.05 vs. control group, ^#^*p* < 0.05 vs. CUMS group

To determine the alteration in miR-29a/b/c-3p in the rat brain after ketamine (10 mg/kg) treatment, qRT-PCR was performed. The results showed that miR-29b-3p was increased in the PFC of the normal rats (Fig. 1b), but did not significantly change in the hypothalamus (Fig. 1a) or hippocampus (Fig. 1c). However, there was no significant change in miR-29a/c-3p in the PFC, hypothalamus, or hippocampus in the normal rats (Fig. 1b). These results indicate that miR-29b-3p is probably a ketamine-sensitive miRNA, especially in the PFC of normal rats.

### miR-29b-3p level in the PFC was decreased in the CUMS rats and restored by the ketamine treatment

To further explore the alteration in miR-29b-3p in the hypothalamus, hippocampus, and PFC of depressed rats, CUMS was utilized to establish a rat depression model. In the behavioral tests, the rats exhibited depressive-like behaviors on the sucrose preference test (*F*_(3,18)_ = 27.710, *p* < 0.001) (Fig. 1d) and FST (*F*_(3,18)_ = 30.741, *p* < 0.001) (Fig. 1e). Subsequently, the hypothalamus, hippocampus, and PFC of the depressed rats were isolated, and miR-29b-3p expression was analyzed by qRT-PCR. The data indicated that miR-29b-3p was decreased by 21.6% (*t* = 4.463, *p* = 0.004) in the PFC of the CUMS rat, but no significant changes were observed in the hypothalamus (*t* = 2.143, *p* = 0.065) or hippocampus (*t* = 2.149, *p* = 0.064) (Fig. 1f). Compared with the saline-treated rats, after the ketamine administration, miR-29b-3p expression increased by 56.7% (*t* = −4.541, *p* = 0.002) in the PFC of the CUMS rats (Fig. 1g). These results revealed that ketamine is able to promote miR-29b-3p expression in the PFC of CUMS rats, suggesting that miR-29b-3p is a possible key factor involved in ketamine’s antidepressant effects.

### miR-29b-3p directly targeted GRM4

**Fig. 2:**
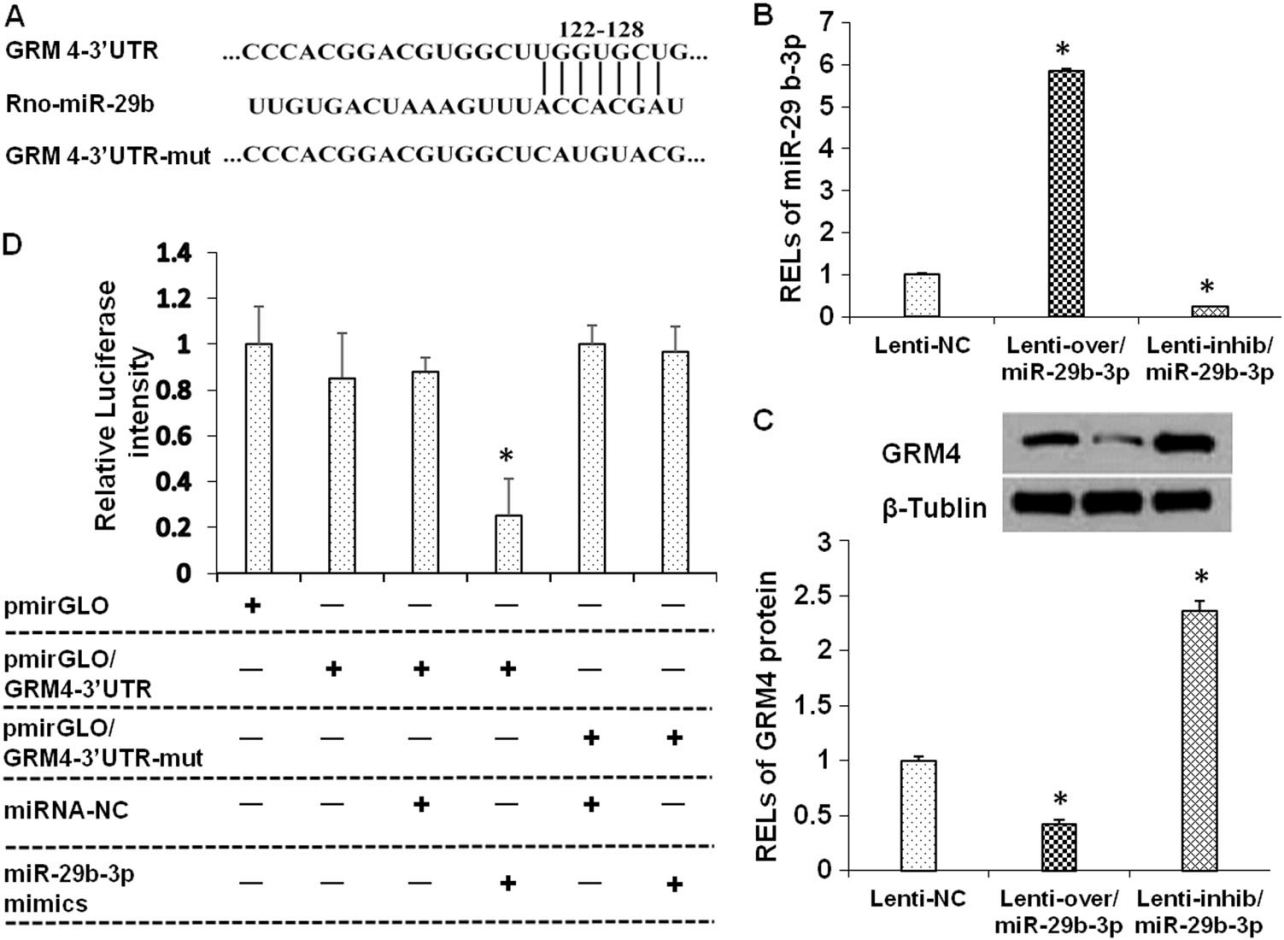
miR-29b-3p directly targets GRM4. miR-29b-3p and GRM4 3′-UTR base pairing at a binding site and the sequence of mutant GRM4 3′-UTR (**a**). RELs of miR-29b-3p in the prefrontal cortex neurons 72 h after lenti-over and lenti-inhib/miR-29b-3p infection (**b**). RELs of GRM4 protein in the prefrontal cortex neurons 72 h after lenti-over and lenti-inhib/miR-29b-3p infection (**c**). Luciferase activities in C6 cells 48 h after transient co-transfection with 50 nM rno-miR-29b-3p mimics or control mimics and 0.5 µg pmirGLO containing wild-type or mutated GRM4 3′-UTR (**d**). RELs relative expression levels. The data represent three sets of independent experiments and are shown as the means ± SD. *p < 0.05

miRNAs have been previously found to regulate distinct genes widely involved in neurodevelopmental and brain disorders^35^. Therefore, we searched for the target genes of miR-29b-3p. The gene targets of miR-29b-3p were predicted using the following four miRNA target prediction databases: MicroCosm, miRanda, TargetScan, and miRDB. The results showed that GRM4 had a putative miR-29b-3p-binding site mapped to the 3′-UTR and was one of the high-scoring candidate genes for miR-29b-3p targeting (Fig. 2a). To test the effect of miR-29b-3p on GRM4 expression, lentiviruses overexpressing and silencing miR-29b-3p (named lenti-over/miR-29b-3p and lenti-inhib/miR-29b-3p) were used to infect primary neuron cells. miR-29b-3p was highly expressed after the lenti-over/miR-29b-3p infection and downregulated after the lenti-inhib/miR-29b-3p infection (Fig. 2b). The expression of GRM4 in the infected cells was analyzed by Western blotting, and the results showed that GRM4 was negatively regulated by miR-29b-3p (Fig. 2c). These findings indicate that miR-29b-3p directly targets GRM4.

To further investigate whether miR-29b-3p directly targets GRM4, luciferase reporters containing either a wild-type GRM4 3′-UTR or a mutant GRM4 3′-UTR and mutant sequences of the miR-29b-3p-binding site were used (Fig. 2a). The results showed that compared with the control group, miR-29b-3p inhibited luciferase activity through the 3′-UTR of miR-29b-3p (Fig. 2d). The reporter activity of the GRM4 3′-UTR containing a mutation in the miR-29b-3p-binding site did not significantly differ from that in the control transfected cells (Fig. 2d).

### Ketamine decreased GRM4 expression in primary neuron cells

**Fig. 3:**
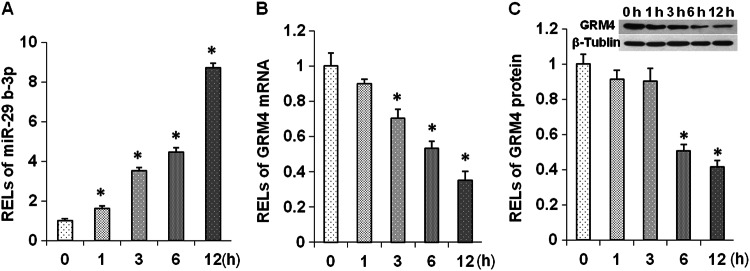
Change in miR-29b-3p and GRM4 levels at different times after ketamine treatment in cultured prefrontal cortex neurons. RELs of miR-29b-3p (**a**) and GRM4 mRNA (**b**) in the prefrontal cortex neurons 1, 3, 6, and 12 h after a 50 µM ketamine treatment. RELs of GRM4 protein in the prefrontal cortex neurons 1, 3, 6, and 12 h after a 50 µM ketamine treatment (**c**). RELs relative expression levels. The data represent three sets of independent experiments and are shown as the means ± SD. *p < 0.05

GRM4 has been implicated in the regulation of anxiety-related behaviors^19^ and MDD^23^. However, whether ketamine influences the expression of GRM4 remains unknown. To validate the effect of ketamine-regulating GRM4 expression, rat PFC primary neuron cells were cultured. After the ketamine treatment, the qRT-PCR analysis showed that the GRM4 mRNA levels gradually declined at 1, 3, 6, and 12 h (Fig. 3b), while the miR-29b-3p levels simultaneously increased (Fig. 3a), indicating that the miR-29b-3p levels were negatively correlated with GRM4 expression. The GRM4 protein levels were also detected by Western blotting and showed a trend similar to that of the mRNA levels (Fig. 3c). These results indicate that GRM4 expression in primary neuron cells is influenced by ketamine in a time-dependent manner.

### Ketamine mitigated the upregulation of GRM4 in the PFC of CUMS rats

**Fig. 4:**
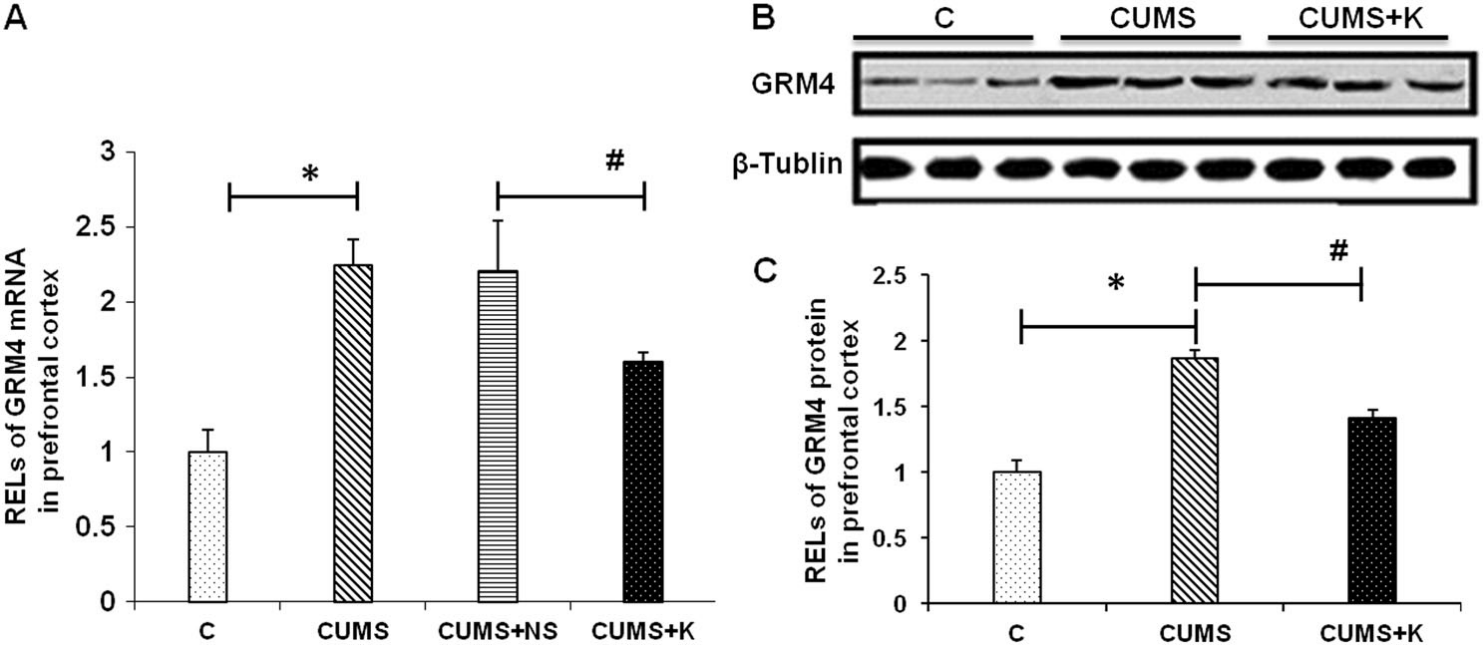
Change in GRM4 expression levels in the prefrontal cortex of depressed rats following ketamine treatment. RELs of GRM4 mRNA in the prefrontal cortex of depressed rats 24 h after a 10 mg/kg ketamine treatment (**a**). RELs of GRM4 protein in the prefrontal cortex of depressed rats 24 h after a 10 mg/kg ketamine treatment (**b**, **c**). C control group, CUMS chronic unpredictable mild stress, CUMS + NS CUMS + 0.9% saline group, CUMS + K CUMS + ketamine group, RELs relative expression levels. The data represent eight sets of independent experiments and are shown as the means ± SD. *p < 0.05 vs. control group, ^#^*p* < 0.05 vs. CUMS group

GRM4 has been reported to be upregulated in the PFC of MDD subjects^36^. Therefore, we examined the function of ketamine in the regulation of GRM4 expression in the PFC of depressed rats. GRM4 expression in the PFC of depressed rats was tested by qRT-PCR and Western blotting after a sub-anesthetic dosage of ketamine. Both the mRNA and protein levels of GRM4 in the PFC were significantly increased in the CUMS rats (vs. the normal rats) and significantly decreased after the ketamine treatment (vs. the saline group) (Fig. 4), suggesting that ketamine could alleviate the upregulation of GRM4 in the PFC induced by CUMS.

### Effects of miR-29b-3p on Ca^2+^ influx and glutamate release

**Fig. 5:**
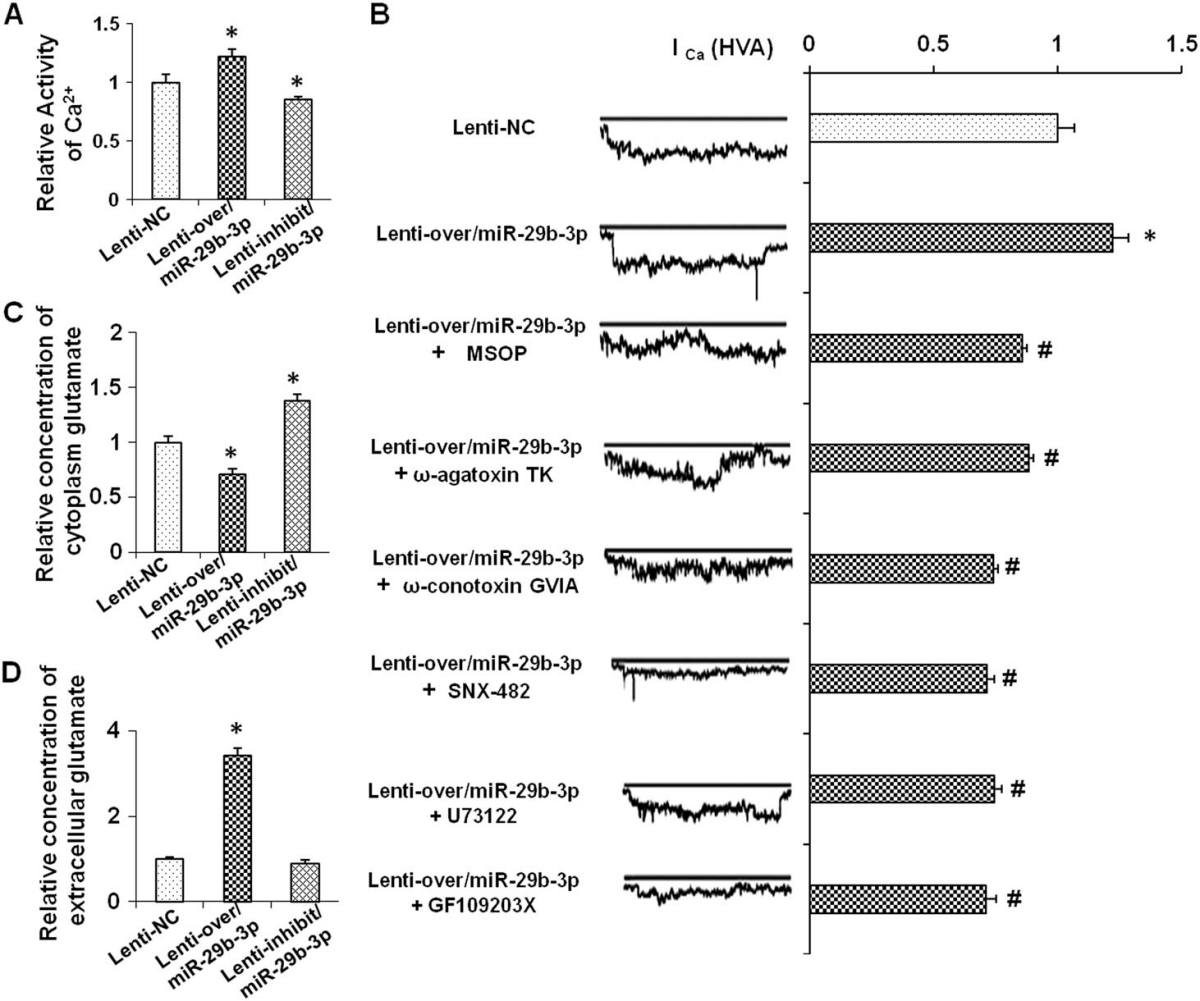
Effects of miR-29b-3p on Ca^2+^ influx and glutamate release. The relative Ca^2+^ activity in the prefrontal cortex neurons 72 h after lenti-over and lenti-inhib/miR-29b-3p infection (**a**). *I*_Ca (HVA)_ current (inward electric current) in cells infected by lenti-over/miR-29b-3p or treated with a GRM4 antagonist (MSOP, 150 μM), a P/Q-type Ca^2+^ channel blocker (ω-agatoxin TK, 250 nM), N-type Ca^2+^ channel blocker (ω-conotoxin GVIA, 250 nM), R-type Ca^2+^ channel blocker (SNX-482, 100 nM), a phospholipase C inhibitor (U73122, 1 μM), or protease C inhibitor (GF109203X, 20 nM) (**b**). Intracellular (**c**) and extracellular (**d**) glutamate concentrations 72 h after lenti-over/miR-29b-3p and lenti-inhib/miR-29b-3p infection. The data represent three sets of independent experiments and are shown as the means ± SD. *p < 0.05 vs. lenti-NC; ^#^*p* < 0.05 vs. lenti-over/miR-29b-3p

Glutamate is a major excitatory neurotransmitter in the mammalian brain. Group III metabotropic glutamate receptors (mGluRs), including GRM4, negatively regulate Ca^2+^ influx into presynaptic terminals^37^ and thus decrease glutamatergic transmission^38,39^. To elucidate the effect of miR-29b-3p on Ca^2+^ influx, Ca^2+^ influx was examined by fluorometric calcium imaging and whole-cell patch-clamp recordings. As shown in Fig. 5a, the downregulation of miR-29b-3p by lenti-inhib/miR-29b-3p decreased the intracellular Ca^2+^ content, and the upregulation of miR-29b-3p by lenti-over/miR-29b-3p increased the intracellular Ca^2+^ content. As shown by the electrophysiology analysis, the overexpression of miR-29b-3p significantly enforced the *I*_Ca (HVA)_ currents (Fig. 5b); the pharmacological agonist of mGluR4 had been proved to acutely inhibit the presynaptic calcium (Ca^2+^) influx that ultimately controls glutamate release^40^. Therefore, we used the antagonist MSOP in this experiment. However, unexpectedly, the group III mGluR antagonist (MSOP, 150 μM) treatment in the cells overexpressing miR-29b-3p also inhibited the presynaptic calcium (Ca^2+^) influx (Fig. 5b).

Presynaptic terminal N-type, P/Q-type, and R-type voltage-dependent calcium channels (VDCCs) play an important role in Ca^2+^ influx^37^. The modulating effect of miR-29b-3p on VDCCs was analyzed, and the results showed that P/Q-type, N-type, and R-type VDCC blocker incubation decreased the *I*_Ca (HVA)_ current promoted by the lenti-over/miR-29b-3p infection (Fig. 5b). Meanwhile, PLC-PKC (protein kinase C)^38,39^ is regarded as an important signaling cascade promoting Ca^2+^ influx. To determine the involvement of miR-29b-3p in PLC-PKC signaling, the inhibitors shown in Fig. 5b were used. The results showed that both the PLC and PKC inhibitors decreased the *I*_Ca (HVA)_ current promoted by the lenti-over/miR-29b-3p infection.

Subsequently, the influence of miR-29b-3p on glutamate release was examined. The cytoplasm and extracellular glutamate levels in the lenti-over/miR-29b-3p-infected and lenti-inhib/miR-29b-3p-infected primary neuron cells was analyzed by a Glutamate Assay Kit. Our data showed that the high miR-29b-3p expression decreased the cytoplasm glutamate concentration, while the decreased miR-29b-3p expression showed the opposite effect (Fig. 5c). As shown in Fig. 5d, the extracellular glutamate levels were increased by the miR-29b-3p overexpression and decreased by the silencing of miR-29b-3p. These findings suggest that miR-29b-3p likely promotes Ca^2+^ influx and then increases glutamatergic transmission by targeting GRM4.

### miR-29b-3p promoted cell survival and cytodendrite growth

**Fig. 6:**
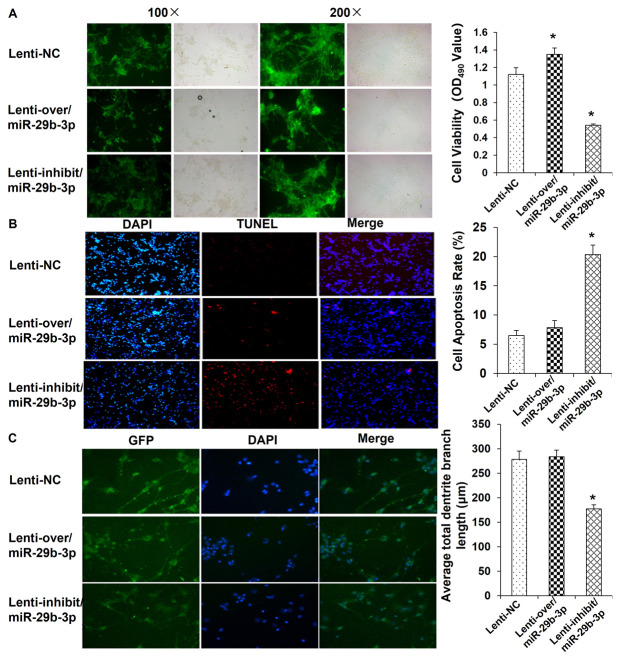
miR-29b-3p promoted cell survival and cytodendrite growth. MTT assay (**a**), TUNEL staining (**b**), and cytodendrite growth observation (**c**) 72 h after lenti-over/miR-29b-3p and lenti-inhib/miR-29b-3p infection. The data represent three sets of independent experiments and are shown as the means ± SD. *p < 0.05 vs. lenti-NC

Brain imaging and postmortem studies suggest that reductions in dendrite arborization and complexity and the number of neurons and glia in brain regions, including the hippocampus, PFC, cingulate cortex, nucleus accumbens, and amygdala, could contribute to depressive symptoms^32–34^. Therefore, we investigated the function of miR-29b-3p in neuron cell survival and cytodendrite growth. The cell viability as determined by an MTT assay was increased by 39% after the lenti-over/miR-29b-3p infection and decreased by 41% after the lenti-inhib/miR-29b-3p infection (Fig. 6a). Cell apoptosis, which was tested by TUNEL staining, was elevated by approximately 3-fold after the miR-29b-3p silencing, but no significant changes were found in the miR-29b-3p-overexpressing cell group (Fig. 6b). Cytodendrite growth was observed with immunofluorescence microscopy and found to be promoted in the lenti-over/miR-29b-3p-infected neuron cells, while no significant changes were found in the lenti-inhib/miR-29b-3p-infected neuron cells (Fig. 6c). These results indicate that miR-29b-3p benefits cell growth, cell survival, and cytodendrite growth.

### miR-29b-3p overexpression improved depressive behaviors in CUMS rats

**Fig. 7:**
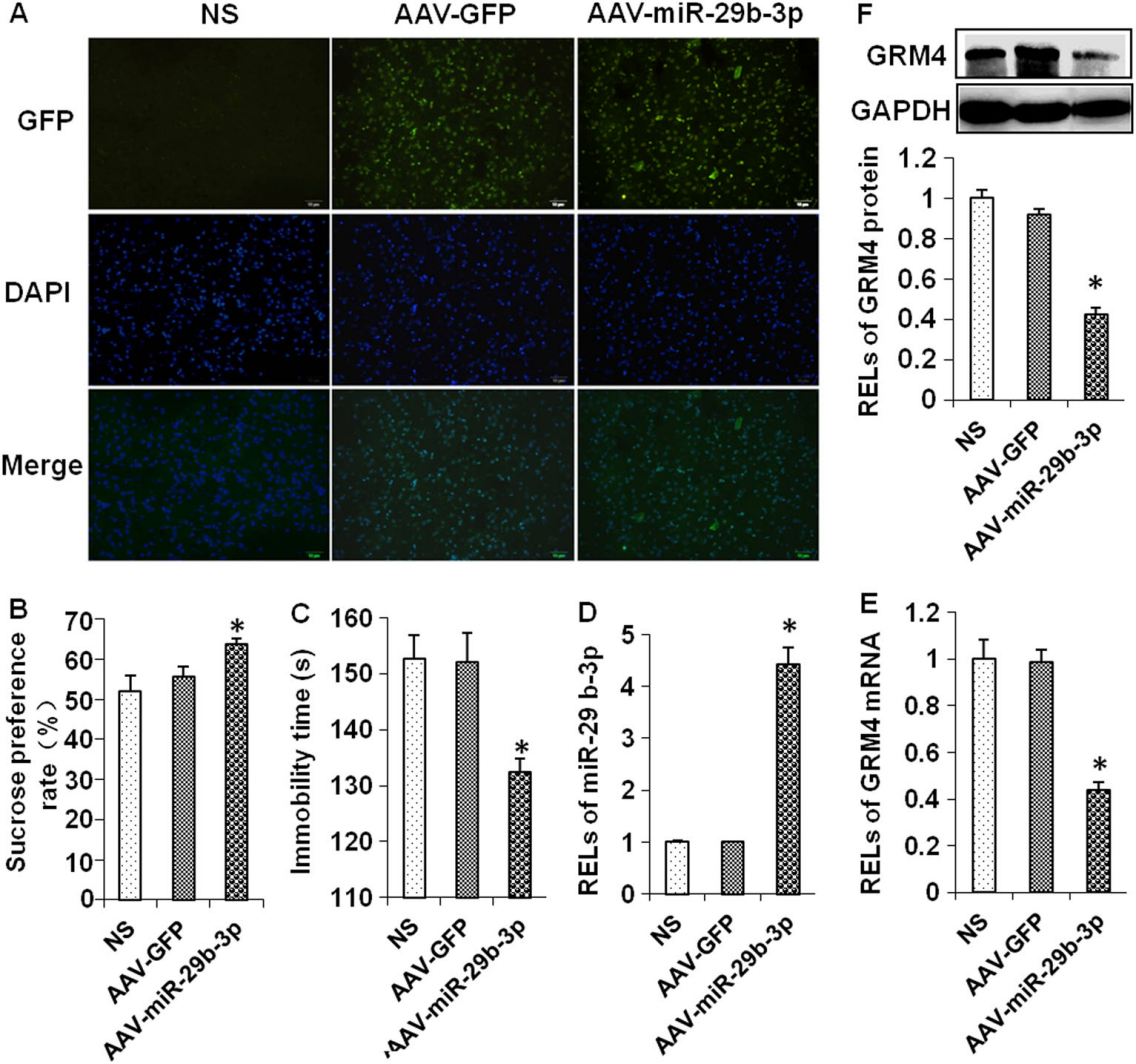
Overexpression of miR-29b-3p improved depressive-like behaviors in CUMS rats. The prefrontal cortex of rats infected by rAAV-miRNA-29b-3p (immunofluorescence microscope) (**a**). The sucrose preference rate (**b**) and immobility time (**c**) of depressed rats 28 days after rAAV-miRNA-29b-3p infection. RELs of miR-29b-3p (**d**), GRM4 mRNA (**e**), and GRM4 protein (**f**) 28 days after rAAV-miRNA-29b-3p infection. RELs relative expression levels, NS CUMS + 0.9% saline group, AAV-GPF CUMS + rAAV-GFP infection group, AAV-miR-29b-3p CUMS + rAAV-miRNA-29b-3p infection group. The data represent eight sets of independent experiments and are shown as the means ± SD. *p < 0.05 vs. rAAV-GFPs

To further determine whether miR-2b-3p could target GRM4 in rat PFC tissue and mitigate the depressive behaviors of depressed rats, AAV-miR-29b-3p was injected into the PFC of CUMS rats using a stereotaxic frame. After 28 days, the AAV effectively infected the brain tissue as indicated by immunofluorescence microscopy (Fig. 7a). Saline and AAV-GFP served as controls.

To examine the effects of miR-29b-3p on major depression disorder, AAV-miR-29b-3p-infected depressive-like rats were subjected to behavioral tests, including a sucrose preference test, open-field test, and FST. The AAV-miR-29b-3p infection led to increased sucrose preference (*F*_(2,15)_ = 32.466, *p* < 0.001) (Fig. 7b) and shortened immobility time in the FST (*F*_(2,15)_ = 58.113, *p* < 0.001) (Fig. 7c), indicating that miR-29b-3p has an antidepressant effect.

The RT-qPCR analysis showed that the level of miR-29b-3p dramatically increased after the AAV-miR-29b-3p infection to 4.42 times that in the AAV-GFP group (Fig. 7d), while GRM4 expression decreased by 56.1% (Fig. 7e). The GRM4 protein levels in the rat PFC were also detected by Western blotting and showed a significant decrease after the AAV-miR-29b-3p infection (Fig. 7f).

## Discussion

Our study uncovers a novel signaling pathway involved in the ketamine-induced alterations in MDD, implicating both upstream and downstream effectors of miR-29b-3p. The miR-29b-3p/GRM4 pathway is a critical mediator of ketamine’s antidepressant function in depressive-like rats, and the miR-29b-3p/GRM4 pathway could be considered a potential therapeutic target, opening up novel avenues for drug design for MDD treatment.

MDD is a prevalent central nervous system disorder associated with differential brain regions, including the hypothalamus, hippocampus, and PFC^32–34^. According to recent research, ketamine has a rapid and persistent antidepressant effect^3–5^ and influences the hypothalamus^41^, hippocampus^18^, and PFC^42^. O’Connor et al.^11^ found that miR-29b could be upregulated by ketamine treatment in the hippocampus in an early depression rat model. However, in this study, we found a slight, nonsignificant increase in miR-29b-3p in the hippocampus of normal rats after acute ketamine administration and that miR-29b-3p may be more sensitive to ketamine treatment in depressed animals than normal animal. Interestingly, our results demonstrated that miR-29b-3p expression could be upregulated by ketamine in the rat PFC. The miR-29b-3p levels gradually increased 1, 3, 6, and 12 h post ketamine incubation, which is consistent with the rapid and persistent antidepressant effects of ketamine. Hence, the corresponding action of ketamine is achieved through miR-29b-3p, which may be a key factor in ketamine’s antidepressant effects. However, the mechanism by which miR-29b-3p is upregulated by ketamine is still unclear. miR-29 expression has been previously reported to be repressed by nuclear factor-κB (NF-κB) signaling, and NF-κB downregulation resulted in a depression^43^, while ketamine could suppress NF-κB activity^44^; therefore, ketamine possibly upregulates miR-29b-3p by inhibiting NF-κB activity. Then, CUMS was used to establish a depression model, and miR-29b-3p expression was analyzed by qRT-PCR. Interestingly, the results showed that miR-29b-3p was decreased in the PFC of the depressive rats, whereas after the ketamine administration, miR-29b-3p was increased in the PFC of the depressive rats. Therefore, miR-29b-3p is involved in depression and ketamine’s antidepressant effects.

However, the downstream mechanism of miR-29b-3p in the progression of depression and ketamine’s antidepressant effects remains unclear. Using the target prediction databases MicroCosm, miRanda, TargetScan, and miRDB, we found that GRM4 may be the downstream target gene of miR-29b-3p. The luciferase reporters also confirmed that miR-29b-3p directly targets GRM4 and that GRM4 was negatively regulated by miR-29b-3p. In addition, Lopez et al.^36^ and Li et al.^20^ found that GRM4 was increased in PFC samples and blood samples from depressed patients, and GRM4 has also been implicated in the regulation of anxiety-related behaviors^19^. Therefore, GRM4 was regarded as a potential therapeutic target gene. Although there is no direct evidence that GRM4 modulates depression-like behaviors based on studies investigating GRM4-overexpressed or GRM4-knockdown animal models, GRM4 has been implicated in the regulation of MDD and is considered an attractive target for drug discovery. In our future research, we aim to further study the function of GRM4 in modulating depression-like behaviors and mediating the antidepressant effects of ketamine in GRM4-overexpressed or GRM4-knockdown animal models. However, whether GRM4 participates in the antidepressant activity of ketamine is unclear. In our study, GRM4 expression was increased in the PFC of depressed rats established by CUMS, and the GRM4 levels were decreased in both the PFC and primary neuron cells after the ketamine treatment, supporting the assumption that GRM4 is a ketamine-sensitive gene. Concomitantly, miR-29b-3p was upregulated by ketamine, negatively correlated with the GRM4 levels, and predicted to be a direct regulator of GRM4, which was further supported by the luciferase reporter assay. miR-1202^36^ and miR-355^20^ have also been found to regulate GRM4 expression, but whether ketamine treatment alters the expression levels of these miRNAs requires further investigation. Based on the miRNA array analysis in our previous study^18^, multiple miRNAs are aberrantly expressed in the rat hippocampus after ketamine administration, and these miRNAs did not include miR-1202 or miR-355. Whether miR-1202 and miR-355 in the PFC and other brain regions respond to ketamine treatment has not been previously studied. In this study, we overexpressed miR-29b-3p in the PFC of depressed rats using the AAV infection technique and determined that miR-29b-3p downregulated the expression of GRM4, suggesting that miR-29b-3p regulates GRM4 expression both in vitro and in vivo. Therefore, GRM4 plays an important role in the downstream mechanism of miR-29b-3p in the progression of depression and ketamine’s antidepressant effects.

miR-29b-3p is involved in the antidepressive-like actions of ketamine, but to strengthen this main conclusion, additional data should be obtained by examining whether the antidepressive-like effects of ketamine are affected in animal models in which miR-29b-3p is overexpressed or knocked down in vivo. Therefore, miR-29b-3p-overexpressing and miR-29b-3p-knockdown animal testing will be performed in the future.

### miR-29b-3p contributes to Ca^2+^ influx in a GRM4-dependent manner

l-Glutamate is a major excitatory neurotransmitter in the central nervous system and activates both ionotropic and metabotropic glutamate receptors. Glutamatergic neurotransmission is involved in most aspects of normal brain functioning and can be perturbed in many neuropathological conditions. GRM4 belongs to metabotropic glutamate receptor group III and is mainly located on the presynaptic membrane, inhibiting Ca^2+^ influx into presynaptic terminals and, thus, decreasing glutamatergic transmission^40^. We examined the effect of miR-29b-3p on Ca^2+^ influx in this study. The calcium imaging showed that miR-29b-3p overexpression increased the intracellular Ca^2+^ concentration, and the whole-cell patch-clamp recording showed that miR-29b-3p overexpression promoted *I*_Ca_ currents. These results indicate that the overexpression of miR-29b-3p targeting GRM4 contributes to Ca^2+^ influx. However, unexpectedly, the MOSP treatment of cells overexpressing miR-29b-3p also inhibited the influx of presynaptic calcium (Ca^2+^). One reason may be that MOSP is a selective antagonist of group III mGluRs, which include the following four subtypes: mGlu4, mGlu6, mGlu7, and mGlu8; compared to mGlu4, the other subtypes probably show different effects on calcium (Ca^2+^) influx. For example, mGlu7 and mGlu8 mediated opposite effects in several studies^45,46^.

The types of presynaptic voltage-gated calcium channels (VGCCs) modulated by miR-29b-3p require further exploration. Our data showed that the overexpression of miR-29-3p increased the Ca^2+^ influx, but was totally inhibited by any type of VGCC, indicating that miR-29-3p is probably involved in VDCC-mediated Ca^2+^ influx, which is consistent with the modulating effect of GRM4 on multiple types of voltage-gated Ca^2+^ channels^47^. The PLC or PKC inhibitor could reverse the increased Ca^2+^ influx induced by miR-29-3p, suggesting that miR-29-3p could influence Ca^2+^ influx through a PLC-PKC signaling cascade, which is consistent with the role of GRM4 in depressing the presynaptic calcium influx via a PLC-PKC signaling pathway^48^.

Our study also found that miR-29b-3p promotes glutamate release. miR-29b-3p’s promotion of Ca^2+^ influx and glutamate release may explain why the miR-29-3p infection led to improvement in the depressive behaviors of the depressed rats. Additionally, miR-29b-3p was found to benefit neuron cell survival and cytodendrite growth in the in vitro cultured neuron model, which could be another reason explaining miR-29b-3p’s beneficial effects on depression. The pharmacological effects of ketamine have been proved to increase mammalian target of rapamycin complex 1 signaling by activating threonine kinase and increasing synaptic number and function in the PFC in vivo^49,50^. In this study, the effects of miR-29b-3p in the in vitro cultured neuron model showed similar results. However, whether these effects are GRM4-dependent remains unclear, and further investigation of relevant novel target genes is essential.

The depressive-like behaviors of the CUMS rats were alleviated by the overexpression of miR-29b-3p in the PFC via AAV infection, but the CUMS rats overexpressing miR-29b-3p significantly differed from the normal rats in the behaviors tests (*p* = 0.002) and FST (*p* = 0.007). These results suggest that the overexpression of miR-29b-3p did not totally reverse the depressive-like behaviors of the CUMS rats. Therefore, further studies are required to uncover the complex mechanisms of MDD.

In conclusion, miR-29b-3p was downregulated in the PFC of the depressed rats and upregulated by the ketamine treatment. GRM4 expression was negatively regulated by ketamine and miR-29b-3p. The miR-29b-3p overexpression contributed to Ca^2+^ influx, glutamate release, neuron cell survival, and cytodendrite growth and subsequently improved the depressive behaviors of depressed rats, representing a potential novel molecular mechanism underlying ketamine’s antidepressant effect.

## Electronic supplementary material

Supplementary Materials
