# Correction: Prefrontal cortex miR-29b-3p plays a key role in the antidepressant-like effect of ketamine in rats

**DOI:** 10.1038/s12276-022-00900-2

**Published:** 2022-11-28

**Authors:** Yun-Qiang Wan, Jian-Guo Feng, Mao Li, Mao-Zhou Wang, Li Liu, Xueru Liu, Xiao-Xia Duan, Chun-Xiang Zhang, Xiao-Bin Wang

**Affiliations:** 1 Department of Anesthesiology, The Affiliated Hospital of Southwest Medical University, Luzhou, Sichuan Province People’s Republic of China; 2 Laboratory of Anesthesiology, The Affiliated Hospital of Southwest Medical University, Luzhou, Sichuan Province People’s Republic of China; 3 Department of Intensive Care Unit, The Affiliated Chaoyang Hospital of Capital Medical University, Beijing, People’s Republic of China; 4 Department of Biomedical Engineering, School of Medicine, University of Alabama at Birmingham, Birmingham, AL USA

Correction to: *Experimental & Molecular Medicine* 10.1038/s12276-018-0164-4, published online 29 October 2018

After online publication of this article, the authors noticed an error in the Results section.

In the original article, there was a mistake in Fig. 6A as published. The original Fig. 6A was provided to examine the infection efficiency of the recombinant lentivirus (Lenti-NC, Lenti-over/miR-29b-3p and Lenti-inhibit/miR-29b-3p), in which duplicated images were found among the Lenti-NC, Lenti-over/miR-29b-3p and Lenti-inhibit/miR-29b-3p groups. The authors have provided new versions of Fig. 6A. The corrections will not affect the results and scientific conclusions of the article.
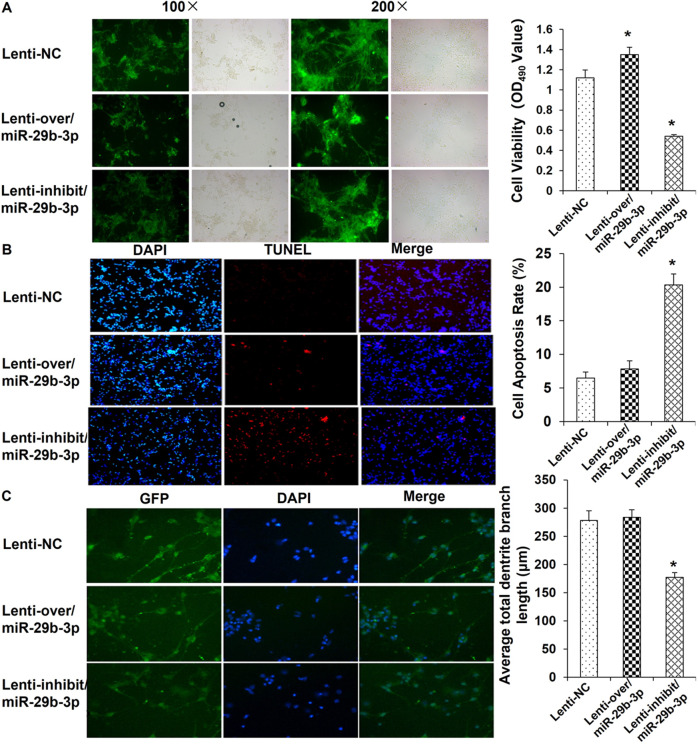


The authors apologize for any inconvenience caused.

The original article has been corrected.

