# Supplementary material for: Prefrontal cortex miR-29b-3p plays a key role in the antidepressant-like effect of ketamine in rats
Source: Exp Mol Med. 2018 Oct 29;50(10):1–14. doi: 10.1038/s12276-018-0164-4 (PMC6204429; doi:10.1038/s12276-018-0164-4)
Supplement: Supplementary file 1 — Supplementary Materials [file 12276_2018_164_MOESM1_ESM.doc]

Supplemental figure. Sequence of pre-miR-29b-3p

CACCTGCTGGATGAGGCCAGCATAGGAGCGCCACCTTTCCCCTCTGTAGGGGACCGTTGGAAGAGGACGAGAACAGACAAAGCTTCTTCAGGAAGCTGGTTTCATATGGTGGTTTAGATTTAAATAGTGATTGTCTAGCACCATTTGAAATCAGTGTTCTTGGTGGAGAACAACTTCGCTGCCGACCACACAAAAGGAAGGAGTGGACAGCCCTGAGGTATTCAGTATCTAAGACAGTCAGGCCACCAGAACCTGCTCAAGTAGCA

The sequence underline is the steam loop of miR-29b-3p.
